# Sensorineural hearing loss in acromegalic patients under treatment

**DOI:** 10.1590/S1808-86942012000400018

**Published:** 2015-10-20

**Authors:** Marcelo Alexandre Carvalho, Renan Magalhães Montenegro Júnior, Marcos Rabelo de Freitas, Lúcio Vilar, Alessandra Teixeira Bezerra de Mendonça, Renan Magalhães Montenegro

**Affiliations:** 1 Otorhinolaryngologist; MSc in Public Health - Federal University of Ceará; 2 Endocrinologist. PhD; Adjunct Professor - Medical School of the Federal University of Ceará; 3 Otorhinolaryngologist; PhD; Adjunct Professor - Medical School of the Federal University of Ceará; 4 Endocrinologist. PhD; Adjunct Professor - Medical School of the Federal University of Pernambuco; 5 Speech and Hearing Therapist - Department of Otorhinolaryngology Walter Cantídio University Hospital - Federal University of Ceará)

**Keywords:** acromegaly, evoked potentials, auditory, brain stem, hearing loss conductive, hearing loss, sensorineural

## Abstract

Acromegaly is a rare endocrine disease. Few studies have evaluated its association with hearing loss (HL) and the results are conflicting.

**Aim**: To evaluate the prevalence and features of HL in a group of patients being treated for acromegaly. To analyze peripheral and central auditory transmission.

**Methods**: Cross-sectional study. A group of 34 patients with acromegaly were submitted to metabolic evaluation, tonal audiometry and brainstem auditory evoked potentials. HL was considered when pure tone average was > 25 DBHL for low frequencies (250, 500, 1000 and 2000 Hz) or high frequencies (3000, 4000, 6000 and 8000 Hz). The patients were divided in group A (with HL) and B (without HL).

**Results**: Twelve patients (35.3%) had sensorineural HL (Group A), being 8 bilateral and 4 unilateral. No one had mixed or conductive HL. The prevalence of diabetes/impaired glucose tolerance was similar between the groups. The frequencies 3000, 4000, 6000 and 8000 Hz were the most affected and with a similar pattern in both ears.

**Conclusion**: sensorineural HL was found in 38.9% of cases. Neither clinical nor metabolic differences were noted between the groups, as well as in regards to peripheral and central auditory transmission.

## INTRODUCTION

Acromegaly is a rare endocrine disease, with an incidence of about five new cases per 1 million inhabitants every year^1^. Most of the cases are associated with a GH-secreting pituitary adenoma; which induces the synthesis of the insulin-like-1 growth factor (IGF-1), which is the main mediator of GH metabolic and cellular proliferation actions^1^, ^2^. The main comorbidities associated with acromegaly are: cardiovascular disease, diabetes, systemic blood hypertension, sleep apnea, arthritis and bone metabolism disorders (osteoporosis)^2^, ^3^, ^4^, ^5^. Among metabolic disorders, we may list carbohydrate metabolism disorders, such as diabetes mellitus, fasting glucose intolerance, insulin resistance, besides reduction in total cholesterol and increase in triglycerides^3^, ^5^.

So far, very few studies have shown the involvement of peripheral nerves and acroparesthesia; nonetheless, without reports of changes to the central nervous systems, except for the compressive changes caused by pituitary tumors^2^, ^5^, ^6^. The involvement of hearing in acromegaly has been studied by numerous authors, with inconsistent results so far.

Maj et al.^7^ mentioned briefly a high incidence of conductive and mixed hearing loss in a group of 34 acromegalic patients.

Menzel^8^ reported the case of a 72-year-old patient with bilateral sensorineural hearing loss with acromegaly, and such finding was associated to a temporal bone hypertrophy, with a resulting narrowing of the internal auditory meatuses, and pressure on the acoustic nerves.

In a study with 15 patients, Richards^9^ found conductive hearing loss in five of the 30 ears investigated, associating this loss with otosclerosis, because in two cases they did unilateral exploratory tympanotomy, in which the stapes was fixed and was replaced by the prosthesis. In these cases, histopathological analysis of the footplates removed, showed active otosclerosis in one patient and inactive in another. Upon analyzing the auditory thresholds of the entire group and comparing it to a control population, stated that there was sensorineural hearing loss in varied degrees in 100% of the patients. There was no statistically significant association between disease duration or circulating levels of GH, although the results seemed to indicate that the hearing thresholds were worse in those patients with lower GH circulating levels.

In a controlled study with 56 individuals with acromegaly referred to pituitary surgery, Doig & Gatehouse^10^, dis not find significant differences between the groups in relation to the auditory thresholds by bone and air conduction.

Crosara et al.^11^ held a controlled study with a group of 15 patients (five men; 10 women; ages between 39-67 years) in which they used tonal audiometry, immittance measures and brainstem auditory evoked potentials (BAEP), and radiological studies of the temporal bone. Results showed a high rate of abnormal results in all frequencies tested, especially at 4 and 8 KHz. Only one patient had mixed hearing loss. As to the BAEP, only 11 patients were assessed, and two had altered results. In both cases, wave I latency was normal in both sides, while in one patient there was an extension of the I-III interval, and in another, intervals I-III and III-V were extended. The radiological assessment did not show changes to the middle ear, optical capsule or internal acoustic meatus. There was no correlation between the degree of hearing loss and GH levels.

Ozata et al.^6^ assessed the central and peripheral nervous systems using BAEP in acromegalic patients. Moreover, they assessed somatosensory evoked potentials of the tibial and median nerves in a group of 10 acromegalic patients (nine men, one woman, aged between 21-65 years) with untreated active disease (GH higher than 1 ng/mL), compared to 20 healthy controls. Patients with changes in the glucose tolerance test of hypothyroidism were taken off the study. Finally, they showed significant extension in the median and tibial nerve potentials; nonetheless, all BAEP components were normal. Thus, the results suggest that there is peripheral nerve involvement without central nerve involvement in patients assessed.

Babic et al.^12^ assessed a group of 30 untreated acromegalic patients, compared to a group of 20 patients in searching for evidence of conductive hearing loss. There was a higher prevalence of middle ear ventilation problems in acromegalic patients, seven (23%), compared to none in the control group, ***p*** = 0.033. Such patients were significantly older, with a longer disease duration and lower medium levels of growth hormone in comparison to acromegalic patients without this problem.

Pilecki et al.^13^ held a study with 37 acromegalic (22 women, 15 men; mean age of 51.7 years, varying between 21.1-77.8 years) patients, compared to 47 healthy controls. They assessed peripheral nerve transmission, represented by the latency of the BAEP wave I, and the transmission at the level of the brainstem, represented by the I-V interpeak. In none of the cases they found alterations to the peripheral transmission. When the 74 traces were analyzed, they found normal interpeak I-V latency results in 34 cases (45.9%), latency extension in 33 cases (44.6%) and shortening in seven cases (9.5%). Such results suggested a non-homogeneous influence of acromegaly on brain function. The authors also suggested that the extension found in I-V intervals could suggest a reduction in the myelination of the auditory pathways of the brainstem, probably because of changes in glucose metabolism present in acromegalic patients. In the study, 12 patients had active disease, while the others were in its inactive stage; however, they did not describe the treatment success used to qualify these groups.

Aydin et al.^14^ published recently the results from functional and structural assessment of hearing in a group of 44 patients. Besides the audiometry and impedance measures, they also carried out MRI of the ear. The patients were further divided into three subgroups according to disease activity, following criteria from Giustina et al.^15^. The mean tonal thresholds (pure tone average-PTA) was determined based on the levels of air conduction in each ear, in the frequencies of 500; 1,000 and 2,000 KHz. PTA > 15 dBHL was considered a hearing loss. Following such criterion, 21 patients (48%) had hearing loss in at least one ear. Moreover, four patients (9%) had conductive hearing loss, 13 (30%) sensorineural, and eight (18%) had mixed hearing loss in at least one of the affected sides. There were no differences between the subgroups as to hearing loss prevalence.

We can clearly see that the conclusions about HL in acromegalics are contradictory. The scarcity of studies and the limited number of patients are determining factors in these assessments. Thus, this paper aims at assessing the prevalence and possible factors associated with the HL in a group of patients with acromegaly in treatment, as well as checking the functioning of the peripheral and central hearing transmission in this group.

## METHODS

We carried out a contemporary cohort study with a cross-sectional cutting in a specialized ward from a tertiary reference center. After approval by the Ethics Committee of the Institution (protocol # 065.08.07), the patients were assessed after signing the informed consent forms.

Sensorineural hearing loss (SNHL) was assessed by means of air and bone conduction pure tone audiometry with the *Beltone 2000 - Clinical Audiometer* (Eletronics corp.; Chicago, Ill.). HL was defined when the mean of the low/medium frequencies (250, 500, 1 and 2 KHz) or the mean in the high tones (3, 4, 6 and 8 KHz) was > 25 dbHL in at least one ear.

Patients with past history of using ototoxic drugs, noise exposure, brain trauma or a past family history of hearing loss were taken off the study. We only included patients with strictly normal otoscopy. Only one patient had a unilateral tympanic perforation and was not included in the group. Two other patients were taken off, because the BAEPs were not possible to acquire because of electrical interference of muscle activity. Two other patients with sensorineural HL were taken off for being 70 years old or older - because of the probable association with presbycusis.

Our final population was made up of 34 patients with acromegaly, 11 men (43.9 ± 6.2) and 23 women (48.7 ± 14.6).

Twelve patients (35.3%) had SNHL, while 22 (64.7%) had normal auditory thresholds. The patients were broken down into two subgroups: group A with SNHL and group B without SNHL. No patient had conductive or mixed HL.

The groups were compared vis-à -vis clinical and laboratory parameters, including serum levels of growth hormone - GH and Insulin-like growth factor - 1 IGF-1. We considered the body mass index (BMI) high when greater than 25 Kg/m^2^.

We also grouped the patients as to the presence of active acromegaly. The patients were divided into two groups: with active disease and controlled disease. We defined controlled disease when the IGF-1 level was normal for gender and age and the GH level < 1.0 μg/litter, as per proposed by Giustina et al.^4^.

Moreover, all the patients were assessed by means of Brainstem Auditory Evoked Potentials (BEAP) with the Medical PC equipment, from Interacustics A/S (Denmark, 2005) with the *IaBaseII, version 4.08* software, in a silent room. The potentials were evoked using 2000 thin clicks, with a stimulation rate of 20 clicks/second, with an intensity of 100 dBHL. The stimuli were monoaural (ABR-3A phone), with contralateral masking (40 dBHL below the stimulus). The band-pass filter was kept between 0 and 3,000 Hz and impedance below 3 Kohms. We assessed the absolute latencies of waves I and the I-III, III-V and I-V interpeak intervals.

In order to assess the distribution normality, we used the Kolmogorov-Smirnov test, when necessary. We used the *t-Student* test to compare the mean values and the *X*^2^ to compare the ratios between groups, as well as the Fisher's exact test. The results are presented as mean ± standard deviation. We established a 5% significance level (α= 0.05). For the statistical analysis, we used the SPSS 12.0 Inc software.

## RESULTS

Twelve (35.3%) patients had sensorineural HL (48.2 ± 9.0 years) - Group A; while 22 (64.7%) had normal hearing threshold (46.6 ± 14.3 years) - Group B. There was no difference vis-à -vis the age between the two groups (*p* = 0.7). Moreover, in both there were similar prevalences of diabetes/glucose intolerance (*p* = 1.5), 41.7% in Group A vs. 63.6% in group B. Group A had equal participation of both genders (six men: six women), while in Group B we found 17 women and five men.

Among the patients in Group A, eight had bilateral HL and four had unilateral. Moreover, four had HL for low and high frequency sounds, while eight had it in the high frequencies only (Table 1).

**Table 1:** Mean low and high frequency pure tone values in patients with SNHL (Group A) in the right and left ears.

Gender	Age (years)	Mean (250, 500, 1 and 2 KHz) DBHL		Mean (3, 4, 6 and 8 Khz) DBHL	
		RE	LE	RE	LE
M	49	17.5	15	35	22.5
M	37	32.5	16.25	23.75	10
M	52	15	15	30	20
M	44	22.5	20	33.75	23.75
M	46	20	15	26.25	27.5
M	40	27.5	36.25	27.5	33.75
F	60	17.5	22.5	48.75	46.25
F	62	22.5	23.75	36.25	28.75
F	48	23.75	22.5	38.75	28.75
F	32	15	15	27.5	20
F	54	23.75	26.25	42.5	38.75
F	55	26.5	33.75	31.25	46.25

RE: right ear; LE: left ear.

**Graph 1:**
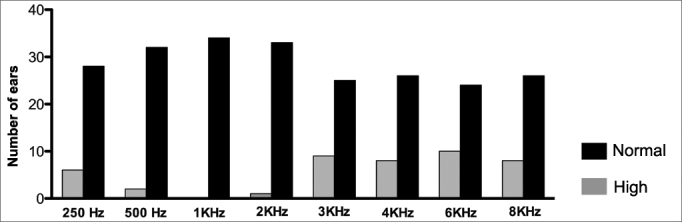
Number of right ears with normal and altered tonal auditory thresholds (> 25 DBHL) in the different frequencies.

**Graph 2:**
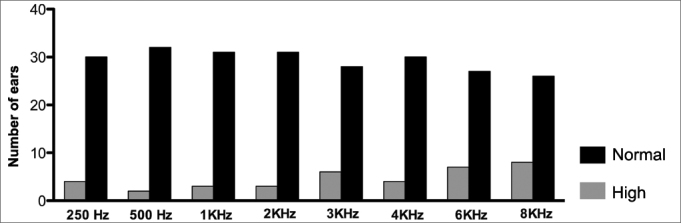
Number of left ears with normal and altered tonal auditory thresholds (> 25 DBHL) in the different frequencies.

When we analyzed each sound frequency alone, we found high auditory thresholds in both ears (> 25 DBHL) in almost all the frequencies analyzed in at least one patient. Such alteration prevailed in the frequencies of 3, 4, 6 and 8 KHz bilaterally (Graphs 1 and 2).

Numerous laboratory data associated with metabolism disorders were assessed in both groups. We assessed the levels of total cholesterol, HDL, LDL, triglycerides, fasting glucose, without significant differences between these parameters in Groups A and B (Table 2).

**Table 2:** Biochemical parameters of patients with acromegaly in Groups A and B.

	Group A	Group B	*p*-value
Glycemia (mg/dL)	96.5 ± 11.9	114.2 ± 28.6	0.5
Total cholesterol (mg/dL)	169.0 ± 49.2	169.4 ± 25.5	0.9
HDL (mg/dL)	44.5 ± 17.3	43.8 ± 11.9	0.8
LDL (mg/dL)	103.6 ± 34.6	92.3 ± 29.6	0.3
Triglycerides (mg/dL)	117.5 ± 79.7	134.1 ± 80.1	0.5
GH (μg/L)	1.7 ± 2.4	4.4 ± 7.3	0.2
IGF1 (ng/mL)	235.2 ± 278.4	313.2 ± 237.9	0.3

Group A patients had lower mean GH levels (mean = 1.76 μg/litter) vis-à -vis group B patients (mean = 4.4 μg/litter), although without significant association (*p* = 0.2). The same happened with IGF-1 levels, when analyzed as continuous variable (Table 2). The mean estimated time of disease duration was 10.2 ± 5.5 years in Group A and 7.7 ± 5.9 years in Group B, without significant differences between the groups (*p* = 0.2).

There was no statistically significant difference between the groups vis-à -vis the categorical variables: systemic arterial hypertension, transsphenoidal surgery, radiotherapy, use of cabergolin, normal IGF-1 level (titrated for gender and age range), normal or high BMI. When the groups were analyzed in relation to active acromegaly and hearing loss, there was no statistical association (*p* = 0.4) When we assessed the wave I absolute latencies and the I-III, III-V and I-V interpeak intervals for the right and left ears, separately, there were no significant differences between Groups A and B (Tables 3 and 4).

**Table 3:** Latencies of the brainstem auditory evoked potentials in the right ears of Groups A and B.

BAEP (ms)	Group A	Group B	*p*-value
Wave I	1.51 ± 0.25	1.39 ± 0.26	0.1
I-III Interval	2.12 ± 0.33	2.27 ± 0.28	0.2
III-V Interval	2.02 ± 0.39	1.91 ± 0.25	0.3
I-V Interval	4.14 ± 0.36	4.19 ± 0.36	0.7

**Table 4:** Latencies of the brainstem auditory evoked potentials in the left ears of Groups A and B.

BAEP (ms)	Group A	Group B	*p*-value
Wave I	1.40 ± 0.20	1.32 ± 0.25	0.3
I-III Interval	2.24 ± 0.33	2.29 ± 0.38	0.6
III-V Interval	1.98 ± 0.28	1.87 ± 0.37	0.4
I-V Interval	4.22 ± 0.31	4.17 ± 0.34	0.7

## DISCUSSION

There are very few papers in the literature discussing the possible association between hearing loss and acromegaly. In the present study, there was a prevalence of 14 patients (38.9%) with sensorineural hearing loss, mainly bilateral and in the high frequencies (Table 1).

Aydin et al.^14^ found a HL prevalence of 48%; 30% sensorineural; however, they used PTA > 15 DBHL as criterion, more sensitive than what was used in the present study.

There were no patients with conductive or mixed HL in the group assessed. Thus, such results did not reinforce what was proposed by Richards^9^ about the higher incidence of otosclerosis in acromegaly, when it is expected to have conductive HL with a normal tympanic membrane.

When we assessed each frequency alone in both ears (Graphs 1 and 2) we also noticed the presence of increased auditory thresholds. Altered results were present in almost all frequencies assessed, at least in one patient. Moreover, they predominated in the high frequency sounds and bilaterally. Such results are similar to those reported by Crosara et al.^11^, who reported high rates of altered results, especially at 4 and 8 KHz.

Upon comparing Groups A and B, it was not possible to identify significant associations between medications being used, comorbidities and metabolism disorders with SNHL. We stress that Group A patients had higher mean age, longer disease durations and lower GH levels than those in Group B; however, without statistical significance. Similarly, Richards^9^ concluded that their results seemed to indicate that the hearing thresholds were worse in those patients with lower levels of circulating GH. Contrary to that, Babic et al.^12^ found similar changes (older age, longer disease duration and lower GH levels) when they compared patients with acromegaly with and without middle ear ventilation disorder; in this case, the changes were statistically significant.

The proportion of patients with active acromegaly, according to criteria from Giustina et al.^4^, was similar in both groups. Thus, there seems not to be, at least directly, negative interference of the excessive levels of GH and IGF-1 on the functioning of the peripheral auditory system, at a cochlear or retrocochlear level.

The present study may have been the first to do the comparison model of the BAEP among acromegalic patients with and without SNHL. When we assessed the transmission of auditory electrical impulses by means of BAEP, we found no significant differences between Groups A and B. All the patients were submitted to checking tonal auditory thresholds and had thresholds which enabled acquiring the potentials.

Thus, even with altered auditory thresholds in different frequencies in Group A, there was no difference between the groups in the neural transmission at the level of the auditory nerve (wave I) and the brainstem (I-III and III-V interpeak interval).

In our study, we could not determine the factors which could be involved in HL development in patients with acromegaly. Associated and prevalent factors in this disease, such as diabetes/fasting glucose intolerance, systemic arterial hypertension and high body mass index were not statistically associated to the outcome. Lower GH levels, longer disease duration and higher mean age may be associated to the sensorineural HL, but require a larger number of studies and larger groups of individuals for a better statistical analysis. Because of this disease rarity, we cannot expect to have studies with larger groups of individuals.

Issues as to the real cause-and-effect relationship between acromegaly and HL require longitudinal studies, in which the causal factors may be assessed with greater accuracy. Cross-sectional studies may reveal statistical associations, however, without determining causality.

## CONCLUSION

In the present study, we found a SNHL prevalence of 35.3% in a group of patients with acromegaly being treated. There was a predominance of bilateral losses and in the high frequencies. The most affected frequencies were 3,000; 4,000; 6,000 and 8,000 Hz, in both ears.

There was no statistical relation between SNHL and the clinical-laboratory parameters analyzed, including disease duration, mean age, disease activity, metabolical disorders, GH and IGF-1 levels. Moreover, there were no peripheral transmission disorder or at the level of the brainstem on the auditory pathways, when Groups A and B were compared.
